# Oxidation-Induced Degradable Nanogels for Iron Chelation

**DOI:** 10.1038/srep20923

**Published:** 2016-02-12

**Authors:** Zhi Liu, Yan Wang, Max Purro, May P. Xiong

**Affiliations:** 1 Pharmaceutical Sciences Division, School of Pharmacy, University of Wisconsin–Madison 777 Highland Avenue, Madison, WI 53705-2222, USA

## Abstract

Iron overload can increase cellular oxidative stress levels due to formation of reactive oxygen species (ROS); untreated, it can be extremely destructive to organs and fatal to patients. Since elevated oxidative stress levels are inherent to the condition in such patients, oxidation-induced degradable nanogels for iron chelation were rationally designed by simultaneously polymerizing oxidation-sensitive host-guest crosslinkers between β-cyclodextrin (β-CD) and ferrocene (Fc) and iron chelating moieties composed of deferoxamine (DFO) into the final gel scaffold in reverse emulsion reaction chambers. UV-Vis absorption and atomic absorption spectroscopy (AAS) was used to verify iron chelating capability of nanogels. These materials can degrade into smaller chelating fragments at rates proportional to the level of oxidative stress present. Conjugating DFO reduces the cytotoxicity of the chelator in the macrophage cells. Importantly, the nanogel can effectively reduce cellular ferritin expression in iron overloaded cells and regulate intracellular iron levels at the same time, which is important for maintaining a homeostatic level of this critical metal in cells.

Iron plays an important role as a cofactor for critical enzymes involved in cellular processes; as such, its homeostatic balance in cells must be tightly regulated^1^. There are presently no effective cures for genetic blood disorders such as sickle cell disease and thalassemia, and first line treatment consist mainly of addressing the symptoms of anemia by repeated blood transfusions over the lifetime of a patient^2^. Since the human body does not have an efficient means of excreting the influx of excess iron, leakage from storage proteins (i.e. ferritin, hemosiderin) and eventual iron saturation of transferrin can ultimately result in the circulation of highly-reactive non-transferrin bound iron (NTBI) localizing to heart and liver cells^3^. NTBI is known to increase cellular oxidative stress levels in cells due to formation of reactive oxygen species (ROS) via the well-known Haber-Weiss reaction^4^. The implications and dangers of excess metals is not only limited to blood disorders because there is also growing evidence that elevated levels of iron and other essential metals may play important roles in disease progression ranging from Alzheimer and Parkinson’s to cancer angiogenesis^5^,^6^,^7^.

Chelation therapy is the most effective way to treat iron overload^8^. The hexadentate metal chelating moiety deferoxamine (DFO) is a drug currently approved by the FDA for treatment of iron overload in patients undergoing prolonged red blood cell transfusions due to anemia^9^. Unfortunately, DFO is currently plagued by toxicity issues, an extremely short residence time in the body (ca. 20 min), and requires constant patient monitoring to avoid excess iron removal. In addition, poor patient compliance is especially problematic, due to the need to infuse DFO over prolonged periods of time, and can result in fatality^10^. In spite of the wide-applicability of chelation therapy to many diseases characterized by metal surpluses, there have only been a handful of publications advancing this area over the years. For example, an upgrade has been to improve DFO pharmacokinetics by conjugating it to linear polymers and dendrimers^11^,^12^,^13^. Although conjugating DFO to polymers has prolonged drug circulation, safe and effective chelating materials for chelation therapy should also possess features capable of regulating iron chelation levels to avoid removing too much iron too fast from cells, oxidation-induced degradable properties suitable for eliminating iron-bound chelates from the body, and an overall lower drug cytotoxicity profile; such favorable properties for iron chelation therapy have not been suitably addressed with previous macromolecular designs.

Recently, cyclodextrin (CD) based supramolecular systems have been utilized to construct various stimuli-responsive materials due to its ability to selectively recognize and form reversible inclusion complexes with different guest molecules^14^,^15^,^16^,^17^,^18^,^19^,^20^. Furthermore, the biocompatibility of CD makes it especially suitable for biological applications^21^,^22^. Herein, we report on a strategy for the preparation of iron-chelating nanogels capable of degrading and chelating at an oxidative stress-dependent rate (oxNG-DFO). Oxidation-sensitive host-guest interactions between β-cyclodextrin (β-CD) and ferrocene (Fc) (Figs S1, S2 and S3) are introduced into the nanogel polymerization cocktail in the form of “host-guest crosslinkers (CL)”^23^ and polymerized in the presence of acrylamide (AAm) and DFO monomers (DFOm) (Fig. S4) by a free radical initiator in reverse emulsion reaction chambers (Fig. 1). The resulting nanomaterial is characterized by DFO moieties for binding to iron and crosslinked by host-guest interactions between β-CD and Fc. In the presence of oxidizing agents such as hydrogen peroxide (H_2_O_2_), the CL holding the nanogel scaffold together begins to degrade at an oxidative stress-dependent rate. This is due to oxidation of the hydrophobic Fc guest to the more hydrophilic Ferrocenium cation (Fc^+^) which significantly reduces its interaction with the β-CD hydrophobic host cavity^24^. The overall synthetic methodology can generate macromolecules in the size range appropriate for drug delivery applications.

## Results and Discussion

### Preparation and Physical Characterization of Nanogels

**Figure 2:**
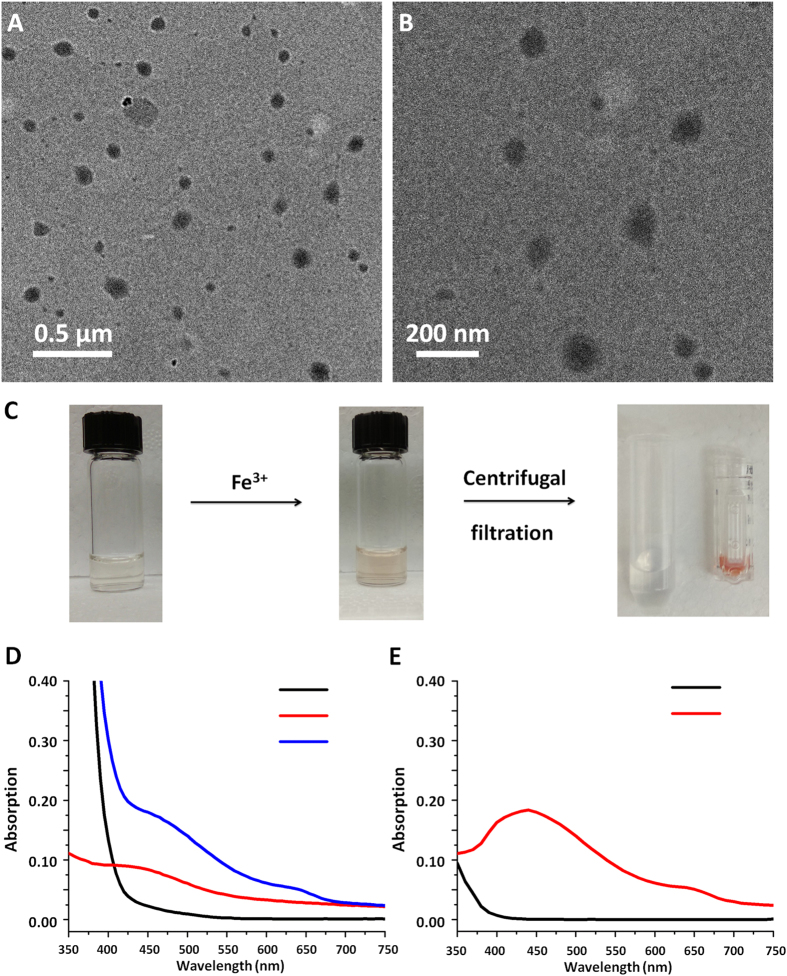
(**A**) Representative TEM image of oxNG2-DFO nanogels at the 0.5 μm scale, and (**B**) at the 200 nm scale. (**C**) Optical images of oxNG2-DFO appear clear in solution, and turns yellow-brown after addition of ferric iron due to formation of the DFO-iron complex. After spin filtering through a centrifugal filter unit (MWCO 10,000), the dark yellow-brown nanogel-iron chelate concentrate cannot pass through the filter. (**D**) UV-Vis absorption spectrum of Fe(III) in solution (black line), oxNG2-DFO in solution (red line), and oxNG2-DFO in the presence of Fe(III) (blue line). (**E**) UV-Vis absorption spectrum of the concentrate displays strong absorption at ca. 430 nm (red line) and no absorption for the filtrate (black line) after extensive washing with the centrifugal filtration unit.

A series of four oxNG-DFOs was prepared in reverse emulsion reaction chambers, by keeping the weight ratio of surfactants used constant, to generate similarly sized nanogels with a z-average diameter of ca. 140 nm (Table 1). The polydispersity index (PDI) of the nanogels ranged from 0.14–0.19, indicating that they were sufficiently monodisperse. By keeping the ratio of CL to AAm to initiator fixed at 1:75:0.3 and varying only the DFOm molar ratio from 5–0.5, DFO density within the final resulting nanogels could be easily controlled. Since the diameter and PDI of nanogels were similar, oxNG2-DFO was used as a representative example for the following study. Representative TEM micrographs of oxNG2-DFO materials are shown in Fig. 2A. At the 200 nm scale level, the oxNG2-DFO averaged ca. 100 nm (Fig. 2B). This is smaller than the size estimated by DLS and may be attributed to nanogel shrinkage during the preparative process of air-drying TEM samples.

To probe the iron chelating capability of nanogels, UV-Vis absorption was used to confirm the formation of a 1:1 complex between conjugated DFO and ferric iron, Fe(III), by monitoring its characteristic absorption peak at ca. 430 nm^25^. After mixing the solution of oxNG2-DFO (0.5 mg/ml) with FeCl_3_ (0.5 mg/ml), a distinct clear yellow-brown color immediately forms, which is indicative of nanogel-iron chelates (Fig. 2C). This was further verified by UV absorbance measurements of oxNG2-DFO/Fe(III) chelates (Fig. 2D). With increasing DFO content in the nanogel series prepared, a deeper yellow-brown color (Fig. S5) and higher absorbance measurements at 430 nm (Fig. S6) were observed, confirming that DFO was indeed successfully incorporated into the scaffold due to formation of increasing iron-DFO complexes.

To further verify that DFO was indeed conjugated to nanogels and not just entrapped, the oxNG2-DFO/Fe(III) mixture was washed extensively with a centrifugal filtration unit (MWCO 10,000) and both the recovered oxNG2-DFO/Fe(III) concentrate and the filtrate were collected. Any free DFO/Fe(III) complex in the mixture would have passed through the filter into the filtrate, but the yellow-brown colored suspension containing chelates remained in the concentrate while the clear solution containing excess iron passed through (Fig. S5). UV absorbance measurements further confirmed these results. As shown in Fig. 2E, the absorption peak at 430 nm was still observable in the recovered yellow-brown solution even after extensive washing but no absorption peak at 430 nm was detected in the filtrate, confirming that DFO was conjugated to the nanogel scaffold.

Atomic absorption spectroscopy (AAS) can be used to directly measure the concentration of iron chelated to nanogels and can simultaneously indirectly measure the percentage of DFO present because DFO binds stoichiometrically with iron at a 1:1 ratio on the order of 10^31^ M^−1^^27^. For this assay, excess FeCl_3_ was incubated with nanogels overnight, and free iron was removed by extensive dialysis. The DFO conjugation levels can be calculated based on known initial and final iron measurements in the sample. Results for all nanogels are summarized in Table 2, with a w/w DFO conjugation level ranging from 2.69–16.49%.

### Degradation Studies

**Figure 3:**
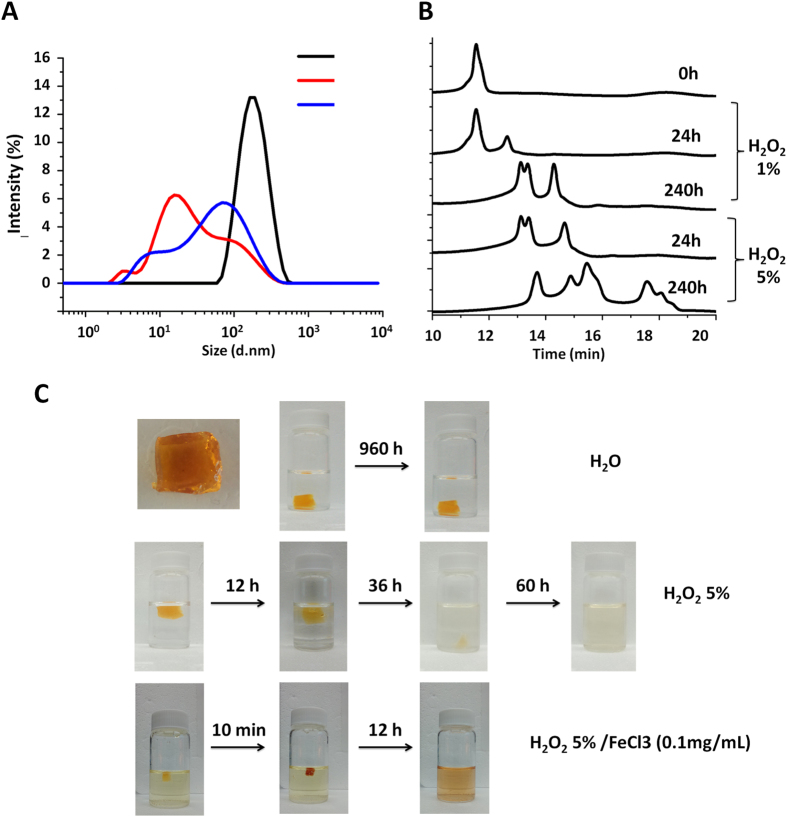
(**A**) DLS size distribution of oxNG2-DFO dispersed in ddH_2_O (black line), 1% H_2_O_2_ (blue line), and 5% H_2_O_2_ (red line) after 240 h incubation at RT. (**B**) Changes in the apparent molecular weight of oxNG2-DFO in the presence of 1% and 5% H_2_O_2_ as monitored by GPC at RT (**C**) Images of oxidation responsive bulk hydrogel slabs incubated under various oxidative stress conditions: (top row) H_2_O_2_ 0%; (middle row) H_2_O_2_ at 5%; (bottom row) H_2_O_2_ at 5%/FeCl_3_ (0.1mg/mL). Hydrogel slab was stable in H_2_O with no significant shape change observed up to 960 h incubation; hydrogel in 5% H_2_O_2_ could no longer be observed with the naked eye after 60 h incubation; in the presence of 5% H_2_O_2_ and FeCl_3_, hydrogel slab visually degraded completely after 12 h incubation. This is likely due to the known catalytic role Fe(III) plays in oxidizing H_2_O_2_ to generate ROS and thus elevating oxidative stress levels even further.

Since oxidative stress levels are typically much higher in patients that are iron-overloaded^26^, oxidation-induced degradation of materials and fine-tuning of the dose provide the most rational mechanisms to tackle both rapid cellular clearance and regulation of iron levels to avoid chelating too much iron. In this study, since the host-guest CL in the nanogel is sensitive to oxidizing conditions, the kinetics of oxidation-induced degradation of oxNG2-DFO was monitored by both DLS and gel permeation chromatography (GPC) up to 240 h (10 days). Since free Fe(III) is known to spontaneously form colloidal-sized particles in aqueous solution which could affect our interpretation of the data, oxNG2-DFO degradation was investigated in the presence of H_2_O_2_ without the metal catalyst. Three different concentrations of H_2_O_2_ without iron were used to simulate different levels of oxidative stress in iron overloaded cell: (a) 0%; (b) 1%; (c) 5%. Interestingly, although the samples were only capped and not stored under an inert atmosphere during these studies, nanogels were relatively stable in ddH_2_O (i.e. 0% H_2_O_2_) and no significant size changes were observed after 24 h or 240 h incubations (Fig. S7A,B). On the other hand, evidence of oxNG2-DFO degradation can be clearly observed after 240 h in the presence of both 1% and 5% H_2_O_2_ at an oxidative stress-dependent rate (Fig. 3A). Z-average diameter of oxNG2-DFO was 136 nm initially but after 24 h incubation in 1% H_2_O_2_, a smaller peak at ca. 20 nm appeared (Fig. S7C). Over the course of 240 h, evidence of further degradation became more apparent by monitoring the PDI of nanogels which increased from 0.15–0.63. Similar to the pattern observed in 1% H_2_O_2_, the z-average diameter of oxNG2-DFO also decreased in 5% H_2_O_2_ at a much faster rate (Fig. S7D) and the PDI increased from 0.15–0.79 by the end of the 240 h study. The DLS data clearly demonstrates that nanogels exhibit varying rates of degradation proportional to the level of oxidative stress. Degradation patterns for oxNG2-DFO were further monitored by GPC (Fig. 3B). The intact nanogels eluted at 11.4 min, but with increased incubation time this peak disappeared and was replaced by peaks with longer elution times indicative of degradation. Increasing the concentration of H_2_O_2_ further increased the degradation rate, as nanogels in 5% H_2_O_2_ at 24 h had a similar GPC curve to the nanogels exposed to 1% H_2_O_2_ at 240 h. After 240 h in 5% H_2_O_2_, more peaks eluting at later times could be observed.

In order to visually confirm the breakdown of the CL, bulk hydrogels with similar compositions to nanogels were prepared and subjected to various conditions to simulate different levels of oxidative stress: (a) H_2_O_2_ 0%; (b) H_2_O_2_ 5%; (c) H_2_O_2_ 5%/FeCl_3_ (0.1 mg/mL). As shown in Fig. 3C, hydrogel was very stable in aqueous solution without H_2_O_2_ and no significant shape change was observed up to 960 h (40 days) incubation. On the other hand, hydrogel in 5% H_2_O_2_ could no longer be observed with the naked eye after 60 h (2.5 days) incubation. In the solution of 5% H_2_O_2_ and FeCl_3_, the degradation rate of the hydrogel proceeded at an even faster rate with the hydrogel completely visually disappearing after only 12 h incubation. These results confirm that elevated oxidative stress levels can indeed dramatically speed up hydrogel degradation rate. In cells, excess iron can catalyze the production of ROS in the presence of H_2_O_2_^26^, and disease states characterized by iron overload conditions do exhibit increased oxidative stress levels which can be particularly advantageous for degradation of the iron-chelating nanogel.

### Cytotoxicity Studies

Although DFO is one of the oldest FDA approved chelators for treatment of iron overload conditions, it possesses undesirable cytotoxic effects and has even been investigated as an anticancer drug in clinical trials for advanced hepatocellular carcinoma^27^,^28^. The cytotoxicity of free DFO and oxNG2-DFO was compared in J774A.1 mouse monocyte/macrophage cells as well as those that had been iron-overloaded with 100 μM ferric ammonium citrate (FAC). J774A.1 macrophage cells were selected for evaluation because excess iron tends to accumulates first in macrophages for storage in ferritin and hemosiderin, so they play an important role in recycling iron under increased catabolism of erythrocytes, a common symptom of anemia-related blood disorders^2^. If iron-overloaded patients are not treated with iron chelation therapy, the continuous supply of surplus iron accumulating with each blood transfusion can eventually overload macrophages and spill into the bloodstream in the form of reactive non-transferrin bound iron (NTBI), resulting in irreparable damage to hepatic cells and other critical organs^10^.

**Figure 4:**
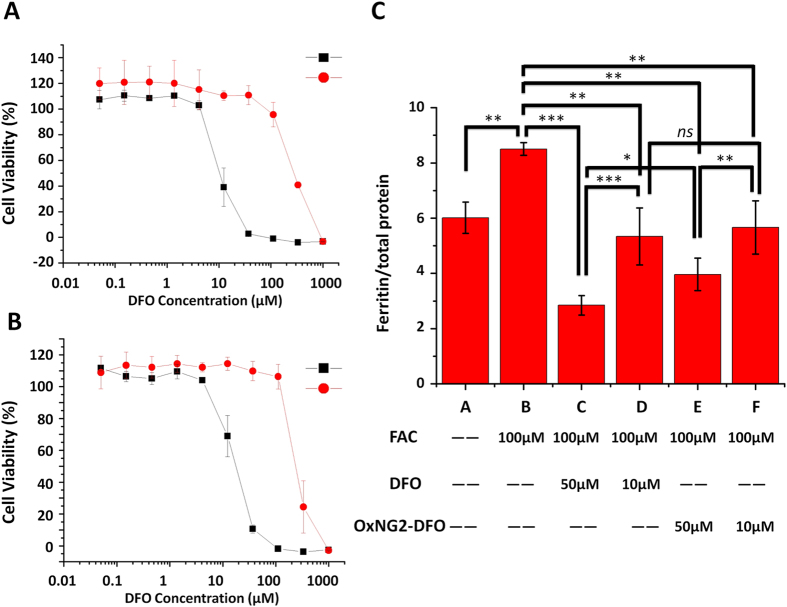
Cytotoxicity of free DFO (black square) and oxNG2-DFO (red circle) on normal J774A.1 cells (**A**) and in iron overloaded J774A.1 cells (**B**) after 48 h incubation; a representative set of data is shown for oxNG2-DFO where each data point is presented as the mean ± SD (n = 3). (**C**) Ferritin reduction assay to monitor iron chelation efficacy of DFO and oxNG2-DFO in iron overloaded J774A.1 cells. Iron overload was induced by 24 h incubation with 100 μM FAC. Cells were then treated with DFO or oxNG2-DFO at 10 μM or 50 μM for 48 h. Cellular ferritin level was measured by a mouse ferritin ELISA assay. Results are normalized to total protein (ng/μg) and presented as mean ± SD (n = 3). “*ns*” means the difference was not significant. *p < 0.05, **p < 0.01, ***p < 0.001.

To evaluate the cytotoxicity of the nanogels, cells in complete DMEM medium were treated with equivalent amounts of free DFO or oxNG2-DFO ranging from 0.05–1000 μM and allowed to incubate for 48 h prior to evaluating cytotoxicity with a metabolism-based resazurin assay. As shown in Fig. 4A, free DFO inhibited 50% viability of normal J774A.1 cells at concentration as low as ca. 10 μM, which is comparable to a previous cytotoxicity report in HUVEC cells^11^. However, oxNG2-DFO was 30-fold less toxic compared to free DFO, with 50% cell viability observed at ca. 300 μM. In Fig. 4B, similar results were obtained in iron-overloaded J774A.1 cells; free DFO inhibited 50% cell viability at ca. 15 μM whereas it took ca. 300 μM oxNG2-DFO to inhibit 50% cell viability. The results demonstrate that conjugating DFO to the nanogels can reduce the cytotoxicity of the chelator.

### Chelation Efficacy in Iron-Overloaded Macrophages

As a proof-of-concept experiment that the nanogels can safely chelate excess intracellular iron, J774A.1 macrophage cells were iron-overloaded with 100 μM FAC for 24 h. The addition of iron in cells results in increased ferritin expression level^29^. We had previously found that 100 μM FAC treatment of cells for 24 h offered the best balance for inducing increased cellular ferritin expression without affecting cell viability (>80% cells were still viable). As shown in Fig. 4C, 100 μM FAC treatment increased cellular ferritin expression from 6.01 ng/μg total protein to 8.51 ng/μg (p < 0.01). Subsequently, iron-loaded cells were treated for 48 h with 10 μM or 50 μM free DFO or equivalent oxNG2-DFO. Free DFO administered at 10 μM was able to reduce cellular ferritin level from 8.51 ng/μg total protein to 5.33 ng/μg total protein (37.4% decrease, p < 0.01), and even further to 2.84 ng/μg total protein (66.6% decrease, p < 0.001) at 50 μM. Treatment with oxNG2-DFO administered at the equivalent of 10 μM DFO decreased ferritin level from 8.51 ng/μg total protein to 5.66 ng/μg total protein (33.5% decrease, p < 0.01), and to 3.63 ng/μg total protein (57.3% decrease, p < 0.01) at the equivalent dose of 50 μM DFO.

Interestingly, this kind of oxNG2-DFO may help regulate iron chelation levels by preventing removal of too much iron from cells. At the lower dose of 10 μM, both free DFO and oxNG2-DFO had similar treatment effects (*ns*) with ferritin returning to non-iron overloaded control baseline level (Fig. 4C, bar A). At the higher dose of 50 μM treatment (bars C and E), ferritin decreased below normal baseline level for both treatments but this effect was less pronounced with oxNG2-DFO compared to free DFO (p < 0.05). Although both 10 μM DFO and equivalent oxNG2-DFO returned iron-overloaded cells to control baseline ferritin levels (bars D and F), there was a drastic difference in cytotoxicity and safety between oxNG2-DFO (>100% cells were viable) and DFO (ca. 50% cells viable) (Fig. 4B). At 50 μM concentration, the cytotoxicity of DFO was even more pronounced, with <50% cells viable compared to >100% cell viability with oxNG2-DFO. The difference can likely be attributed to a combination of iron chelation and cytotoxic properties. For example, a critical difference between free DFO and oxNG2-DFO may relate to the role excess iron plays as a catalyst in the production of ROS. As the rate of oxNG2-DFO degradation correlates directly with oxidative stress levels, the nanogel can respond to its environment and only expose DFO as needed rather than removing too much iron too fast, as is the case with free DFO. As oxidative levels begin to normalize again with reduction of the chelatable iron pool, degradation of the nanogel and hence iron chelation correspondingly slows down. For intracellular chelation, it is undesirable to chelate too much iron from cells since it is a critical cofactor for many enzymes responsible for maintaining cellular function. Therefore, oxNG2-DFO is not only as effective a chelator as free DFO in reducing cellular ferritin level, but also a much safer choice for iron chelation due to its ability to sense intracellular oxidation levels.

## Conclusions

In summary, we have designed and synthesized oxidation-induced degradable nanomaterials capable of chelating iron by incorporating oxidation-responsive host-guest CL and metal chelating drug DFO into the nanogel scaffold. This nanomaterial can degrade at rates proportional to oxidative stress levels in cells, while retaining its iron-chelating properties and exhibiting negligible cytotoxicity. Metal chelators other than DFO (e.g. EDTA and Clioquinol derivatives) may be incorporated into the nanogel scaffold for different applications and it is anticipated that this kind of oxidation-responsive nanogel could find broad applications ranging from various metal chelation therapies in humans to serving as chemical oxidation sensors.

## Methods

### Synthesis of Material Precursors and Preparation of Nanogels

Details of material syntheses and their respective NMRs, and preparation of nanogels are given in Supporting Information.

### Physical Characterization of Nanogels

To investigate the iron chelation capability of the system, UV/Vis absorption spectra of oxNG-DFO with a surplus concentration of Fe(III) was monitored by scanning between 350–750 nm with a SpectraMax Plus spectrophotometer (Molecular Devices). The magnitude of the absorbance peak at 430 nm is characteristic of degree of complexes formed between DFO and Fe (III).

To investigate the morphology of resulting nanogels, transmission electron microscopy (TEM) images were taken on a Tecnai TF-12 instrument with an acceleration voltage of 120 λkV. Sample was prepared by air-drying a drop of 0.01 mg/mL nanogel suspension on copper grid.

To investigate the hydrodynamic size and polydispersity (PDI) of nanogels, dynamic light scattering (DLS) measurements were collected on a Zetasizer Nano ZS (Malvern Instruments, UK) and analyzed with Zetasizer software v7.10. Briefly, nanogels were suspended in ddH_2_O at about 1 mg/mL and the cumulant analysis method was used to calculate the z-average diameter and PDI. Measurements were conducted on three batches of samples and results are reported as mean ± standard deviation (SD). Prior to measurement the nanogel solutions were clarified by filtering through Millipore membranes with a 0.45 μm pore size.

To determine the amount of ferrocene-cyclodextrin crosslinkers present in the nanogel formulation, iron present in ferrocene (Fc) was measured by atomic absorption spectroscopy (AAS). Typically 1 mg of oxNG-DFO was dissolved in 1 mL ddH_2_O and iron content was measured by AAS on a GBC 932AA instrument. The Fc content in oxNG-DFO can then be calculated by equation 1:


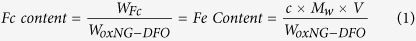


where *c* is the concentration of iron as determined by AAS (mol/L) which should in turn be equivalent to the concentration of Fc, *M*_*W*_ is the molecular weight of Fc (186 g/mol), *V* is the volume of the solution, and *W*_*oxNG−DFO*_is the concentration of oxNG-DFO (mg/mL).

Next, to determine the amount of DFO monomers incorporated into the nanogel scaffold, an excess amount of FeCl_3_ was added to 1 mg/mL nanogel solution and incubated overnight. Afterwards the mixture was extensively dialyzed (MWCO 10,000) against deionized water to remove excess Fe(III) ions. After dialysis, the final volume in solution was measured to account for any dilution effects and Fe(III) concentration was measured by AAS as before. Since DFO chelates Fe(III) at a 1:1 mole ratio and the amount of iron from Fc had already been previously determined, the final DFO content in oxNG-DFO nanogels can be calculated from equation 2:


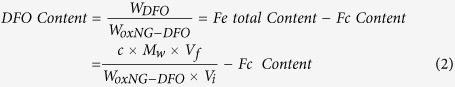


where *c* is the total concentration of Fe(III) as determined by AAS (in mol/L) which should in turn be equivalent to the concentration of DFO, *M*_*W*_ is the molecular weight of DFO (560 g/mol), *V*_*f*_ is the final volume after dialysis, *V*_*i*_ is the initial volume of the solution before dialysis, and *W*_*oxNG–DFO*_is the concentration of oxNG-DFO (mg/mL).

### Degradation Studies

DLS and aqueous phase gel permeation chromatography (GPC) were used to measure the apparent molecular weight changes of oxNG-DFO under varying incubation conditions (eg. 0%, 1%, or 5% H_2_O_2_). Degradation of nanogels via DLS was monitored as described above using a Zetasizer Nano ZS instrument. GPC data acquisition was conducted on a Shimadzu UFLC system equipped with Shodex OHpak SB-806M HQ column (8.0 × 300 mm), and eluted with MilliQ water at a flow rate of 0.5 mL/min. Nanogels were detected with a refractive index detector (RID). GPC data was then analyzed with Shimadzu LCsolution GPC postrun software. For bulk gel degradation studies, the material was scaled up appropriately and then incubated under similar conditions, with the addition of 0.1 mg/mL FeCl_3_ to the 5% H_2_O_2_ solution to demonstrate the catalytic effect of gel degradation under increased oxidative stress conditions. For bulk gel studies, gel degradation was visually assessed.

### Cytotoxicity Studies

Mouse macrophage/monocyte cell line J774A.1 was purchased from American Type Culture Collection (ATCC). Cells were seeded in 96-well plates at a density of 3,000 cells/well, cultured at 37 °C, 5% CO_2_ with DMEM complete medium (supplemented with 10% (v/v) heat-inactivated FBS, 100 I.U./mL penicillin and 100 μg/mL streptomycin), and allowed to settle for 24 h. Cells were then treated with DFO or oxNG-DFO at equivalent DFO concentrations of 1 mM prepared by 1:3 serial dilutions.

Cell viability was measured with the metabolism-based resazurin assay. Briefly, the substrate resazurin was dissolved in cell culture medium at a concentration of 44 μM, added to each well (100 μl) and incubated at 37 °C for 4 h. The fluorescence was measured with excitation at 560 nm and emission at 590 nm, on a SpectraMax Gemini EM microplate reader. Readings from the wells without cells were used as *E*_*blank*_, and the readings from control cells without treatment (*E*_*control*_) represented 100% cell viability. The viability of treated cells at different concentrations can be calculated by equation 3:


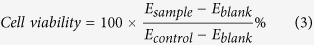


Similarly, cytotoxicity was also evaluated in iron-overloaded J774A.1 cells. Cells were iron overloaded for 24 h prior to the DFO cytotoxicity study by incubation with culture medium containing 100 μM ferric ammonium citrate (FAC) (cells >80% viable with FAC incubation, data not shown).

### Chelation Efficacy in Iron-Overloaded Macrophages

J774A.1 macrophage cells were seeded in 6-well plates at a density of 30,000 cell/well and allowed to settle for 24 h at 37 °C, 5% CO_2_ with DMEM complete medium before treatment. The cells were treated with 100 μM FAC (added to DMEM complete medium) for 24 h to induce iron overload. Subsequently, cells were washed with PBS and treated with DFO or oxNG-DFO at both 10 μM and 50 μM for 48 h. Control group A cells were not iron-overloaded with FAC; cells in control group B were iron-overloaded with FAC but not treated with DFO or oxNG-DFO. After 48 h incubation with DFO or oxNG-DFO, cells were lysed with cell lysis buffer (150 mM NaCl, 10 mM Tris, 1% Triton X-100 and protease inhibitor cocktail, pH 7.4) and total protein concentration was measured with the BCA protein assay kit. Cellular ferritin concentration was measured with a mouse ferritin ELISA kit. The results are plotted as the ratio of ng of ferritin per μg total protein concentration.

### Statistical Analysis

Statistical analysis was performed with GraphPad Prism 5.0 software. Statistical significance between groups was assessed with Student’s t-test; a two-tailed p < 0.05 was considered statistically significant.

## Additional Information

**How to cite this article**: Liu, Z. *et al*. Oxidation-Induced Degradable Nanogels for Iron Chelation. *Sci. Rep*. **6**, 20923; doi: 10.1038/srep20923 (2016).

## Supplementary Material

Supplementary Information

## Figures and Tables

**Figure 1:**
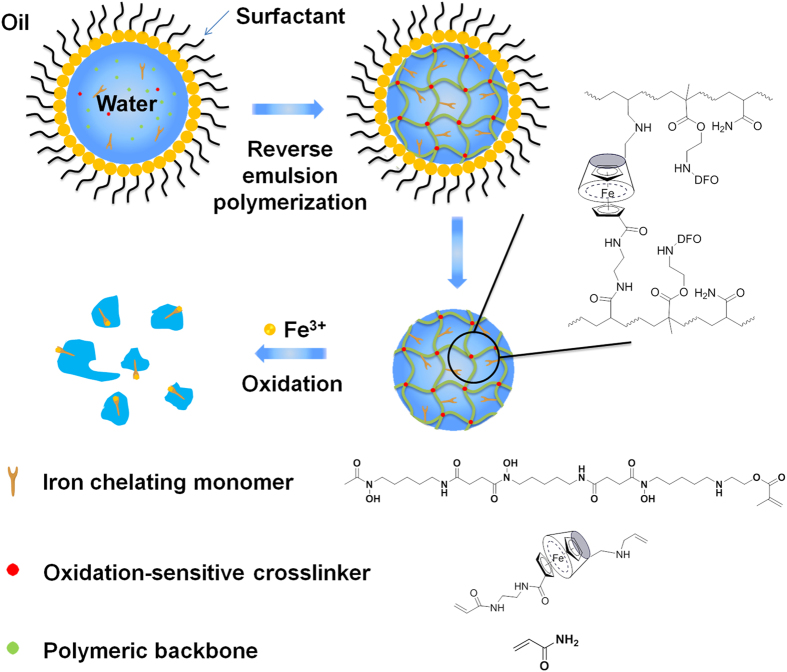
Synthesis of oxidation-sensitive iron chelating nanogels (oxNG–DFO) by free radical polymerization of AAm, oxidation-sensitive crosslinkers (CL) relying on host-guest interactions between β-CD and Fc, and metal chelating deferoxamine (DFO) moieties in reverse emulsion reaction chambers.

**Table 1:** Preparation and characterization of nanogels at different DFO ratios.

	Host-guest CL (mg)	AAm (mg)	VA-044 (mg)	DFOm (mg)	Molar ratio	Z-average diameter (nm)	PDI
oxNG1-DFO	45.7 (1:1)	150	3	100	1:75:0.3:5	148 ± 13	0.14 ± 0.03
oxNG2-DFO	45.7 (1:1)	150	3	50	1:75:0.3:2.5	136 ± 9	0.15 ± 0.02
oxNG3-DFO	45.7 (1:1)	150	3	25	1:75:0.3:1.25	131 ± 16	0.19 ± 0.04
oxNG4-DFO	45.7 (1:1)	150	3	10	1:75:0.3:0.5	141 ± 10	0.16 ± 0.03

The nanogel scaffold was prepared by reverse emulsion polymerization. The reverse emulsion was composed of a continuous phase of hexane and a dispersed aqueous phase stabilized by a mixture of two surfactants, AOT and Brij 30, at the same molar ratio of 1:2.4. Both z-average diameter and PDI were measured by DLS.

**Table 2:** Overall summary of ferrocene (Fc) and DFO content in oxNG-DFO, as measured by AAS.

	Fc (%)	Fc (w/w)	DFO (%)	DFO (w/w)
oxNG1-DFO	66.62	2.99	35.64	16.49
oxNG2-DFO	59.61	2.86	49.32	12.21
oxNG3-DFO	64.26	3.27	45.21	5.92
oxNG4-DFO	55.81	3.00	48.60	2.69

Since Fc consists of two cyclopentadienyl rings bound on opposite sides of a central Fe atom and DFO can complex with Fe(III) at a 1:1 stoichiometric ratio, AAS can be used to directly measure the concentration of Fe present in the nanogels both before and after addition of chelatable iron.

Final Fc and DFO content in oxNG-DFO were then calculated using the equation described in the methods section.
