# Supplementary material for: Oxidation-Induced Degradable Nanogels for Iron Chelation
Source: Sci Rep. 2016 Feb 12;6:20923. doi: 10.1038/srep20923 (PMC4751432; doi:10.1038/srep20923)
Supplement: Supplementary Information [file srep20923-s1.pdf]

Supporting Information

Oxidation-Induced Degradable Nanogels for Iron Chelation

Zhi Liu[1], Yan Wang[1], Max Purro[1], May P. Xiong[1]*

[1]Pharmaceutical Sciences Division, School of Pharmacy, University of Wisconsin–Madison 777 Highland Avenue, Madison, WI 53705-2222, USA

*Correspondence and requests for materials should be addressed to May P. Xiong. E-mail: may.xiong@wisc.edu

Supporting Information

1. Materials

Dioctyl sulfosuccinate (AOT), Brij 30, acrylamide (AAm), glycidyl methacrylate (GMA), sodium periodate ($NaIO_4$), sodium cyanoborohydride ($NaBH_3CN$), ferric chloride hexahydrate ($FeCl_3 \cdot 6H_2O$) were purchased from Sigma-Aldrich (St. Louis, MO). Ferric ammonium citrate (FAC), ferrocenecarboxylic acid (Fc-COOH), *N*-Hydroxysuccinimide (NHS), β-cyclodextrin (β - CD), 1-(3-Dimethylaminopropyl)-3-ethylcarbodiimide hydrochloride (EDC), ethylenediamine (EDA), triethylamine (TEA), sodium hydroxide, p-toluenesulfonyl chloride (TsCl), allylamine and 1,4-dihydroxybenzene were purchased from VWR (Radnor, PA). β-CD was purified by recrystallization from water before use. Deferoxamine mesylate (DFO) was obtained from the University of Wisconsin Hospital Pharmacy Services (Hospira). 2,2'-azobis[2-(2-imidazolin-2-yl)-propane] dihydrochloride (VA-044) was purchased from Wako Pure Chemical Industries, Ltd. Dulbecco's modified eagle medium (DMEM), heat-inactivated fetal bovine serum (FBS), penicillin/streptomycin solution (100×) and the Pierce BCA protein assay kit were purchased from Thermo Fisher Scientific (Waltham, MA). Mouse ferritin ELISA kit was purchased from Immunology Consultants Laboratory (Portland, OR). All other reagents were commercially available and used as supplied without further purification.

2. Synthesis of Material Precursors

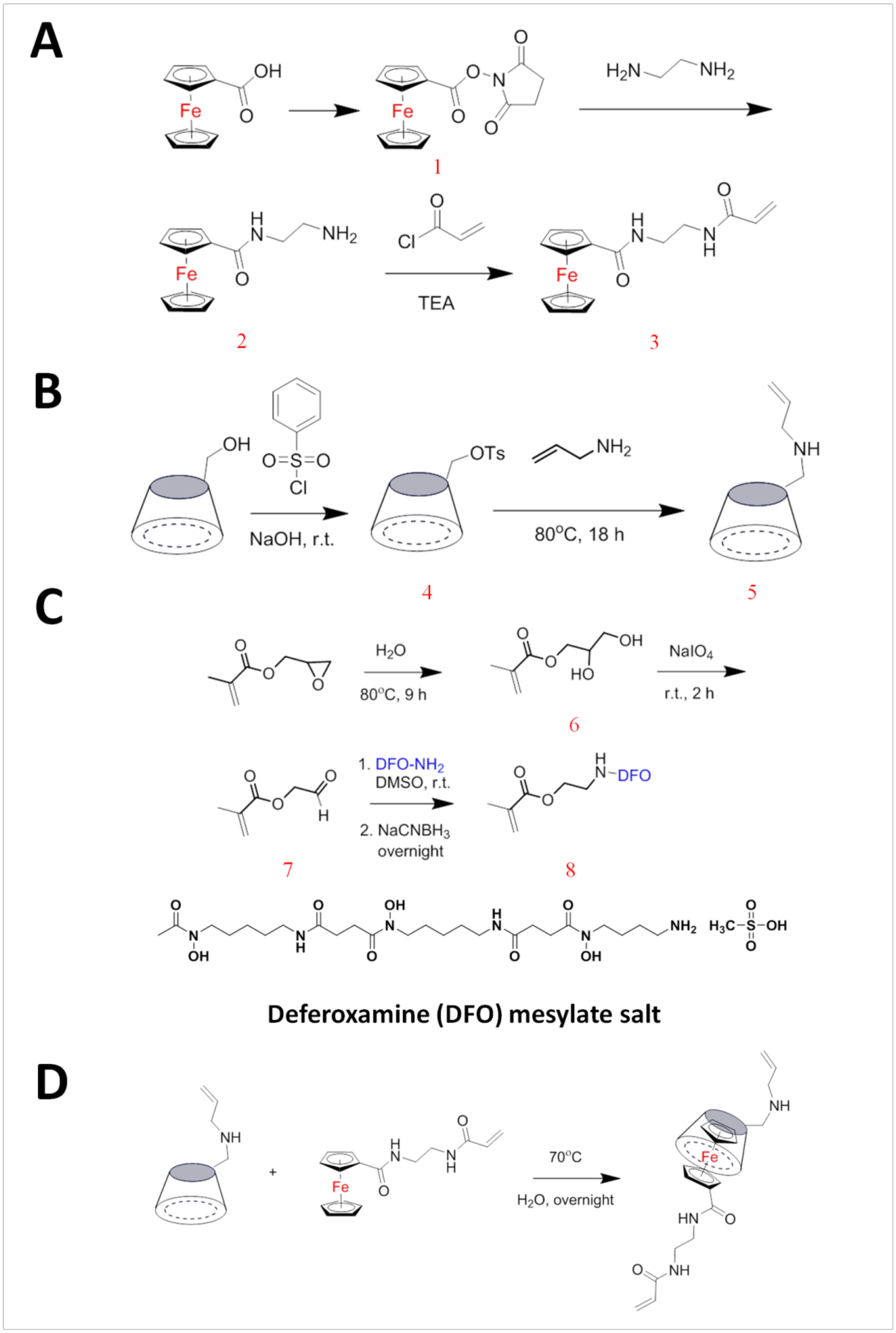

Figure S1. Synthetic route for preparing: (**A**) Fc-AAm (**3**), (**B**) β-CDm (**5**), (**C**) DFOm (8) and host-guest β-CDm:Fc-AAMm crosslinkers.

2-1 Synthesis of Fc-AAm, β-CDm, and DFOm monomers

Fc-AAm (3) (Figure S1A). The precursor Fc-NHS (1) was synthesized following a reported method.[1] Briefly, Fc-COOH (49.9 mg, 0.22 mmol), EDC (58.6 mg, 0.32 mmol), and NHS (37.5 mg, 0.32 mmol) were dissolved in dry DCM (5 mL). The reaction mixture was stirred for 24 h at room temperature. After filtering, the filtrate was concentrated and dried in vacuo. The Fc-NHS was obtained by silica gel column chromatography (dichloromethane:methanol = 10:1) as an orange solid. ^{1}H-NMR of Fc-NHS (400 MHz, $CDCl_3$, 298 K): δ = 2.93 (s, 4H, -CH_2-CH_2-), 4.42 (s, 5H, Cp), 4.60 (m, 2H, Cp), 4.97 (m, 2H, Cp).

Next, Fc-NHS (32.7 mg, 0.10 mmol) was dissolved in 5 mL of DCM; EDA (1 mL, 14.80 mmol) and TEA (1 mL, 7.20 mmol) were also dissolved in 5 mL of DCM. Next, the Fc-NHS solution was added dropwise into the EDA/TEA DCM solution and the reaction mixture was stirred overnight at room temperature. After filtering the reaction, the filtrate was concentrated, followed by washing with water and brine, and dryed over Na_2SO_4. The DCM layer was precipitated with 50 mL of hexane, and the solid product was collected via centrifugation and dried in vacuo to obtain Fc-CONH-$(CH_2)_2$-NH_2 (2) as a yellow powder. Next, Fc-CONH-$(CH_2)_2$-NH_2 (33.7 mg, 0.12 mmol) and TEA (25 μL, 0.18 mmol) were dissolved in THF (2.5 mL). Acryloyl chloride (12 μL, 0.15 mmol) was added dropwise to the reaction while in an ice bath. The reaction mixture was stirred for 2 h at room temperature. After filtering, the filtrate was concentrated and dried in vacuo. The final Fc-AAm product (**Fig. S2**) was obtained by silica gel column chromatography (dichloromethane:methanol = 9:1) as an orange solid. **^{1}H NMR of Fc-AAm** (400 MHz, $CDCl_3$, 298K): δ = 3.56 (t, 4H, Fc-CONH-$(CH_2)_2$-NHCO-), 4.18 (s, 5H, Cp), 4.35 (t, 2H, Cp), 4.71 (t, 2H, Cp), 5.66-5.70 (m, 1H, olefin), 6.13-6.20 (m, 1H, olefin), 6.30-6.35 (m, 1H, olefin), 6.65-6.75 (d, 2H, amide).

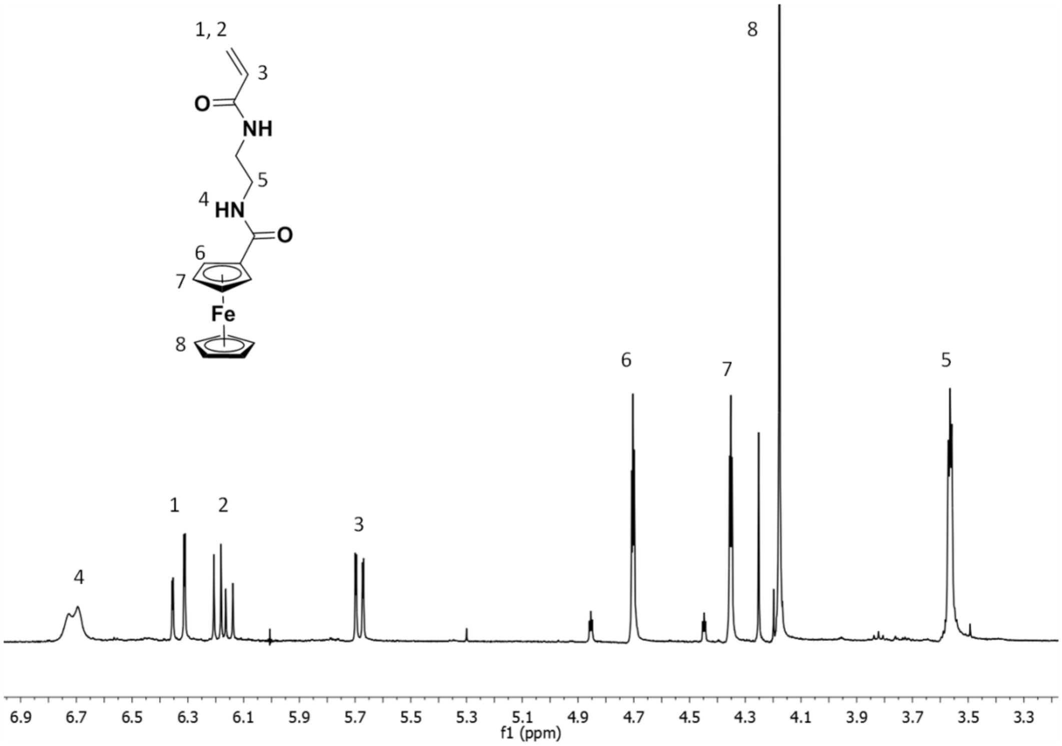

Figure S2. ^{1}H-NMR spectra of **Fc-AAm** (**3**) in $CDCl_3$.

β-CDm, mono-6-(allyl amino)-β-CD (5) (Figure S1B). Mono-6-OTs-β-CD (β-CD-Ts) was first prepared as previously reported.[2] Briefly, dry β-CD (6 g, 5.27 mmol) was dissolved in 50 mL of water. Next, sodium hydroxide (0.657 g, 16.43 mmol) was dissolved in 2 mL of water and added dropwise into the β-CD solution. The mixed solution was immersed into an ice bath. Subsequently, 3 mL of p-toluenesulfonyl chloride (1.21g, 6.35 mmol) in acetonitrile was added in small portions under vigorous stirring over 10 min to the solution. The resulting suspension was stirred for 2h at room temperature and then quickly filtered. The filtrate was refrigerated overnight at 4°C. The resultant precipitate was filtered off, washed three times with water and acetone and recrystallized from hot water three times. The final product was dried under vacuum. ^{1}H NMR of β-CD-Ts (400 MHz, DMSO-d6, 298K): δ = 2.43 (s, 3H, Ph-CH3), 3.15–3.40 (m, H2, H4 overlap with water), 3.40–3.75 (m, 25H, H3, H5 and H6 CyD), 4.15 (m, 1H, H5' CyD), 4.30 (m, 2H, H6' CyD), 4.35–4.50 (m, 6H, OH6 CyD), 4.71–4.80 (m, 7H, H1 CyD), 5.59–5.83 (m, 14H, OH2 and OH3 CyD), 7.43 (d, 2H, Ph), 7.77 (d, 2H, Ph).

Next, β-CD-Ts (1.97 g, 1.53 mmol) was reacted with excess amount of allylamine (30 mL, 306 mol) in the presence of a small amount of 1,4-dihydroxybenzene at 80°C for 18h. After the reaction was completed, the resulting solution was cooled to room temperature and diluted with MeOH (30 mL). When acetonitrile (100 mL) was added, a colorless solid precipitated. The precipitate was collected by centrifugation and repeatedly dissolved in MeOH and poured into a large amount of acetonitrile several times. After filtering and drying under high vacuum, the final product was obtained (**Fig. S3**). **^{1}H NMR of β-CDm** (400 MHz, DMSO-d6, 298K): δ = 3.12 (m, 2H, CyD-NH-CH2-), 3.21-3.72 (m, H2, H3, H4, H5 and H6 CyD); 4.62 (br, 6H, OH6 CyD), 4.75 (s, 7H, H1CyD), 4.93 -4.99 (d, 1H, olefin), 5.05-5.16 (d, 1H, olefin), 5.65-5.71 (br, 14H, OH2 and OH3 CyD), 5.75-5.85 (m, 1H, olefin).

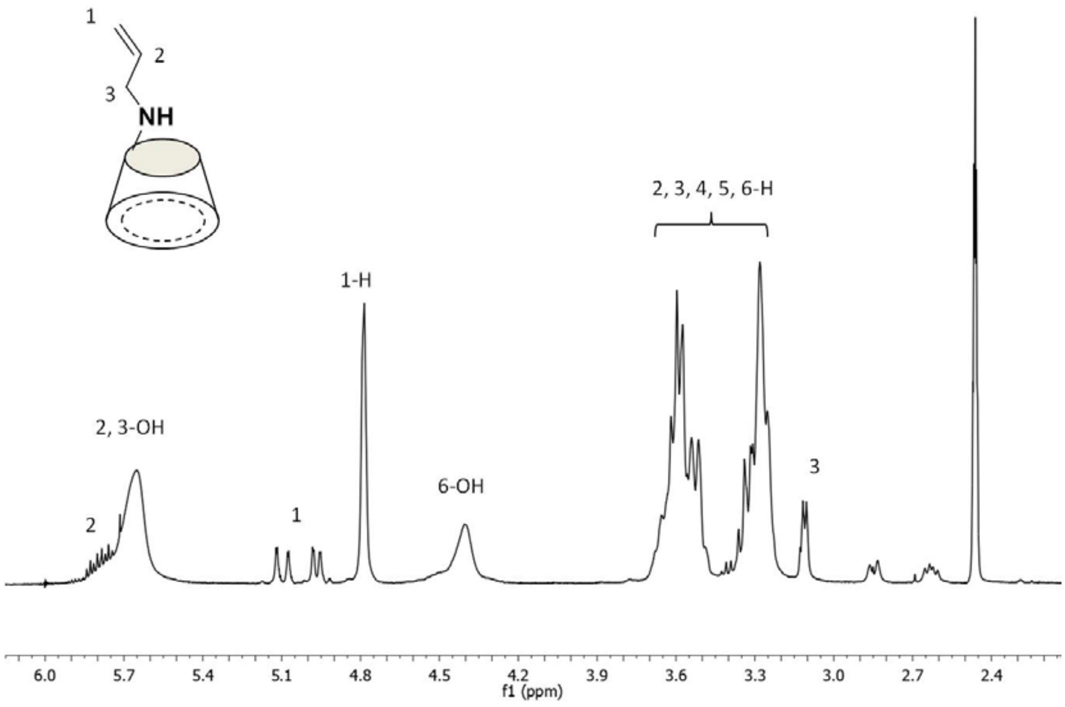

Figure S3. ^{1}H-NMR spectra of mono-6-(allyl amino)-β-cyclodextrin (5), **β-CDm**, in DMSO-d_6.

DFOm (8) (Figure S1C). 1-Glycerol methacrylate (hydrolyzed glycidyl methacrylate, hGMA) was synthesized by hydrolysis of GMA following a reported method.[3] In a typical experiment, GMA (2.63 g, 18.50 mmol) was added to 27 mL deionized water (dH_2O) in a round bottom flask fitted with a condenser and the mixture was stirred and refluxed at 80°C for 9 h. The top of the condenser was open to the atmosphere during the reaction, for the purpose of allowing oxygen to inhibit the polymerization of monomers. The initial emulsion was stirred for 9 h at 80°C and eventually became a homogeneous aqueous solution. ^{1}H-NMR of hGMA (**6**) (400 MHz, D_2O, 298 K): 1.90 (s, 3H, –CH3), 3.56–3.68 (m, 2H, –CH2), 3.72–3.76 (m, 2H, –CH2), 3.94–4.02 (m, 1H, –CH), 4.11–4.28 (m, 2H, –CH2), 4.98-5.04 (m, 1H, –CH), 5.66–5.74 (m, 1H, olefin), 6.09–6.18 (m, 1H, olefin).

Next, $NaIO_4$ (1.34g, 6.26 mmol) was added to 10 mL of hGMA (10% w/w) aqueous solution and the mixture was stirred at room temperature for 2h. The mixture was extracted with DCM and evaporated under reduced pressure. After filtering, the final product was extracted from water with 10 mL of DCM, followed by drying over Na_2SO_4. The DCM layer was concentrated and dried in vacuo. The final 2-oxoethyl methacrylate (OEMA) product was obtained by silica gel column chromatography (dichloromethane:methanol = 1:1) as a colorless oil. ^{1}H-NMR of OEMA (400 MHz, $CDCl_3$, 298 K): 1.90 (s, 3H, –CH3), 4.74 (s, 2H, –CH2), 5.61–5.75 (m, 1H, olefin), 6.13–6.26 (m, 1H, olefin), 9.69 (s, 1H, –CHO).

Finally, DFO mesylate (65.7 mg, 0.10 mmol) and OEMA (19 mg, 0.15 mmol) were dissolved in 2 mL of DMSO and stirred at room temperature for 4h. $NaBH_3CN$ (10 mg, 0.16 mmol) was added to the mixture and stirred overnight. The resultant precipitate was filtered off and the filtrate was added to 20 mL of ether. The precipitate was collected by centrifugation and repeatedly dissolved in MeOH, filtered, and re-precipitated from ether several times. After drying under high vacuum, DFOm (8) was obtained as white solid (**Fig. S4**). **^{1}H-NMR of DFOm** (400 MHz, $CDCl_3$, 298 K): 1.15-1.5 (m, 18H, –CH_2-), 1.85 (s, 3H, –CH3), 1.91 (s, 3H, –CH3), 2.19-2.28 (m, 2H, –CH_2-NH-), 2.30- 2.51 (m, 8H, –CH_2-CO-), 2.51-2.75 (m, 2H, –CH_2-NH-), 2.84-3.00 (m, 4H, –CH_2-NHCO-), 3.25-3.5 (m, 6H, –CH_2-NOH-), 4.12 (t, 2H, -CH2-OCO-), 5.63–5.70 (m, 1H, olefin), 5.94–6.10 (m, 1H, olefin), 7.60-7.75 (br, 3H, -NH), 9.51-9.64 (m, 3H, -OH)

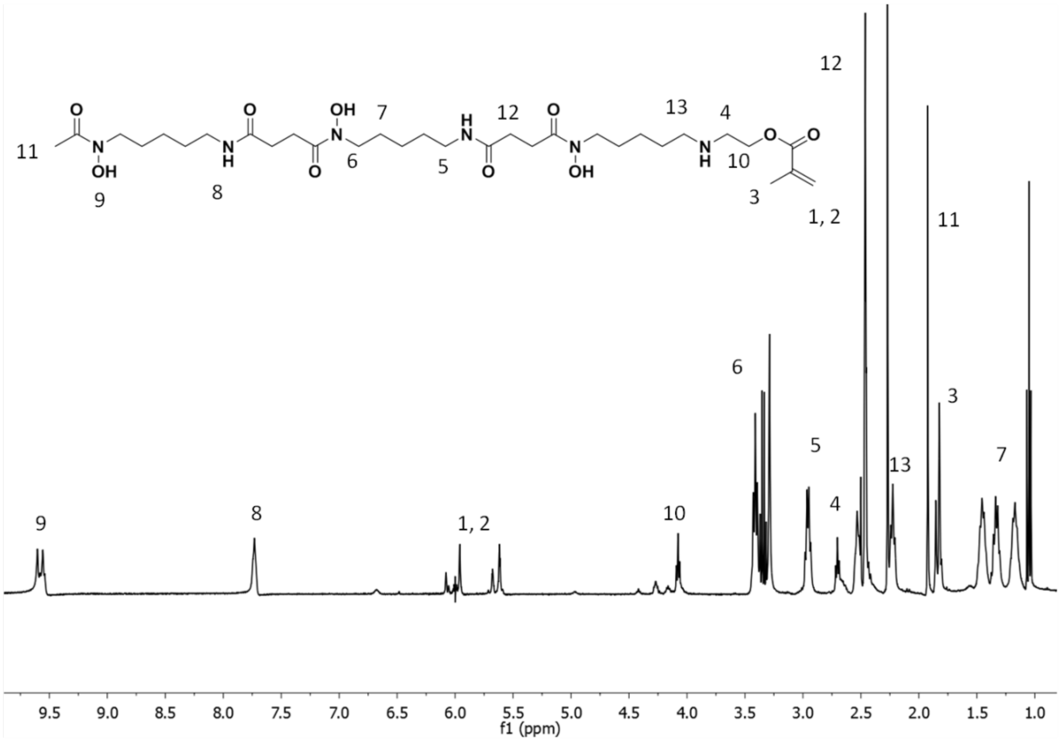

Figure S4. ^{1}H-NMR spectra of **DFOm** (**8**) in DMSO-d_6.

2-2. Preparation of hydrogels and nanogels

The host-guest crosslinkers were first pre-assembled by mixing β-CDm (36 mg, 0.030 mmol) and Fc-AAm (9.8 mg, 0.030 mmol) in ddH_2O (1 mL) with stirring at 70 °C overnight to yield a transparent solution (**Figure S1D**).[4]

To prepare the bulk hydrogels, 150 mg acrylamide was dissolved into 1 mL aqueous solution of host-guest crosslinker. Next, 3 mg VA-044 was added and the solution was purged with nitrogen gas for 1 h. The reaction was heated at 50 °C overnight to yield a gel. The gel was washed repeatedly with water to remove any unreacted molecules. To prepare the various nanogels, the scaffold was synthesized via a modified reverse emulsion polymerization method. To a 50 mL round bottom flask, 0.79 g AOT and 1.54 g Brij 30 were added. Next, 22 mL hexane was added to dissolve the surfactants under magnetic stirring at 1000 rpm. The stirring emulsion was purged with nitrogen gas for 1h to remove dissolved oxygen. Separately, 150 mg acrylamide and varying amounts of DFOm was dissolved into 1 mL aqueous solution of host-guest crosslinker. The precursor solution was purged with nitrogen gas for 1 h and slowly added to the stirring

surfactant/hexane solution with the aid of a syringe to form reverse water-in-oil emulsions. Next, 3 mg VA-044 in 50 μl dH_2O was added to the mixture and heated at 50 °C overnight to initiate polymerization. When the polymerization was completed, hexane was removed on a rotary evaporator, and the nanogel was precipitated with 25 mL methanol, centrifuged, and washed with ethanol 6x (25 mL each) to remove excess surfactants, initiators, unreacted monomers and cross-linkers. Final nanogels were dialyzed (MWCO 10,000) against ddH_2O (six changes within 24h). The final product can be lyophilized to yield a yellow colored solid for prolonged storage.

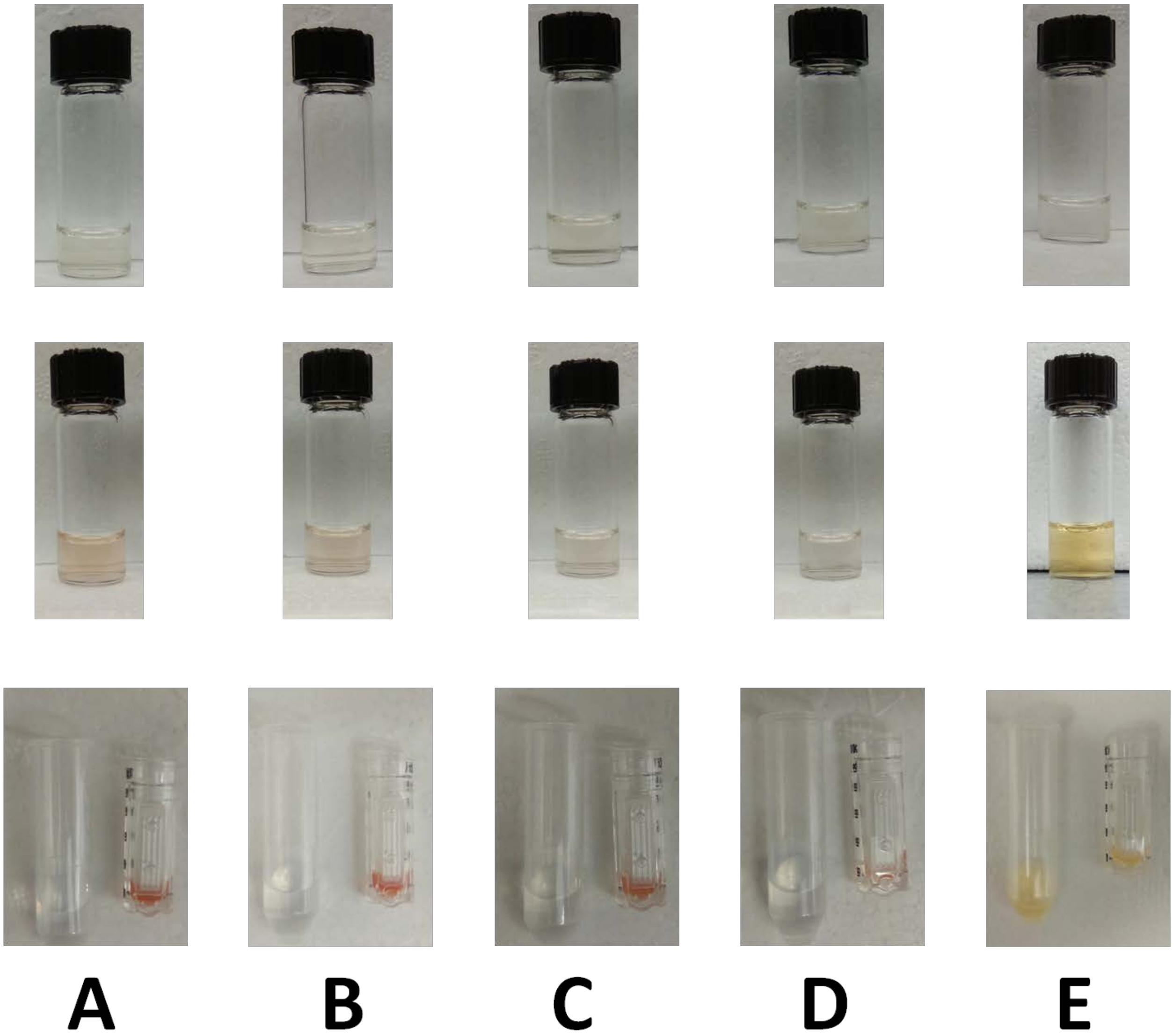

Figure S5. Optical images of oxNG1-DFO (**A**), oxNG2-DFO (**B**), oxNG3-DFO (**C**) oxNG4-DFO (**D**) and DFO (**E**) before addition of iron reveal clear solutions (first row of vials). After addition of iron, a distinct clear yellow-brown color forms immediately and is indicative of nanogel-iron chelates (second row of vials); note that more DFO conjugated to the nanogel correlates with a deeper yellow color in the vials. To further rule out the possibility that DFO was just loosely associated with nanogels, a microcentrifuge filter tube (mwco 10,000 g/mol) was used to concentrate the material (third row of images); the yellow colored suspension in (**A**)(**B**)(**C**)(**D**) containing chelates remained in the filter unit and the clear solution containing excess iron passed through; in contrast to the nanogel-DFO formulations, the yellow free DFO-iron colored suspension in (**E**) passed through the filter into the filtrate.

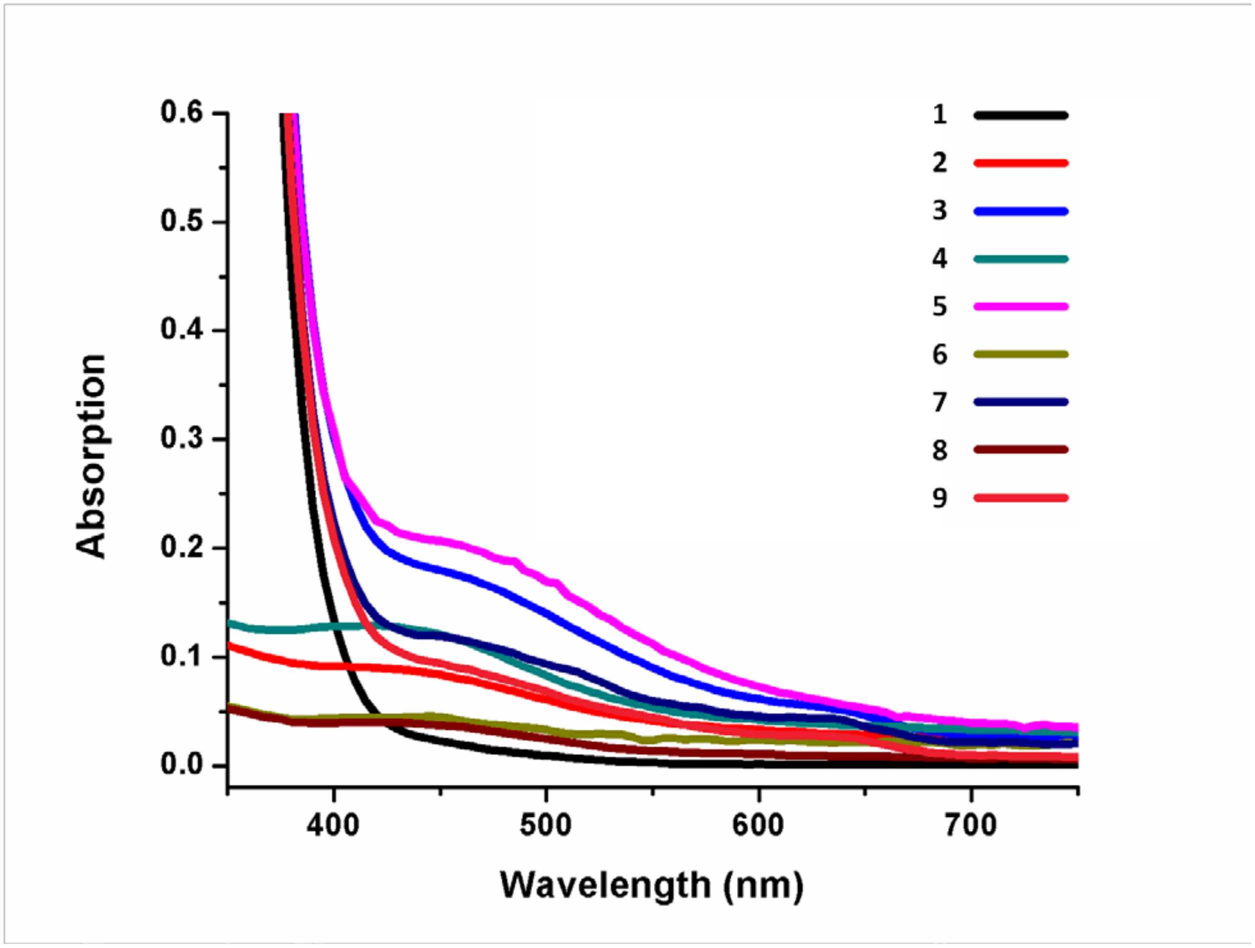

Figure S6. UV-Vis absorption spectrum of Fe(III) (line 1) or nanogels alone reveal only minimal absorbance at ca. 430 nm: oxNG1-DFO (line 4), oxNG2-DFO (line 2), oxNG3-DFO (line 6), oxNG4-DFO (line 8). In contrast, UV-Vis absorption spectrum of nanogel-iron chelates in solution reveal a strong absorption peak at ca. 430 nm: oxNG1-DFO/Fe(III) (line 5), oxNG2-DFO/Fe(III) (line 3), oxNG3-DFO/Fe(III) (line 7), and oxNG4-DFO/Fe(III) (line 9). The increasing absorbance at 430 nm correlates with more complex formation and indicates that DFO at various levels was indeed successfully conjugated to the nanogels.

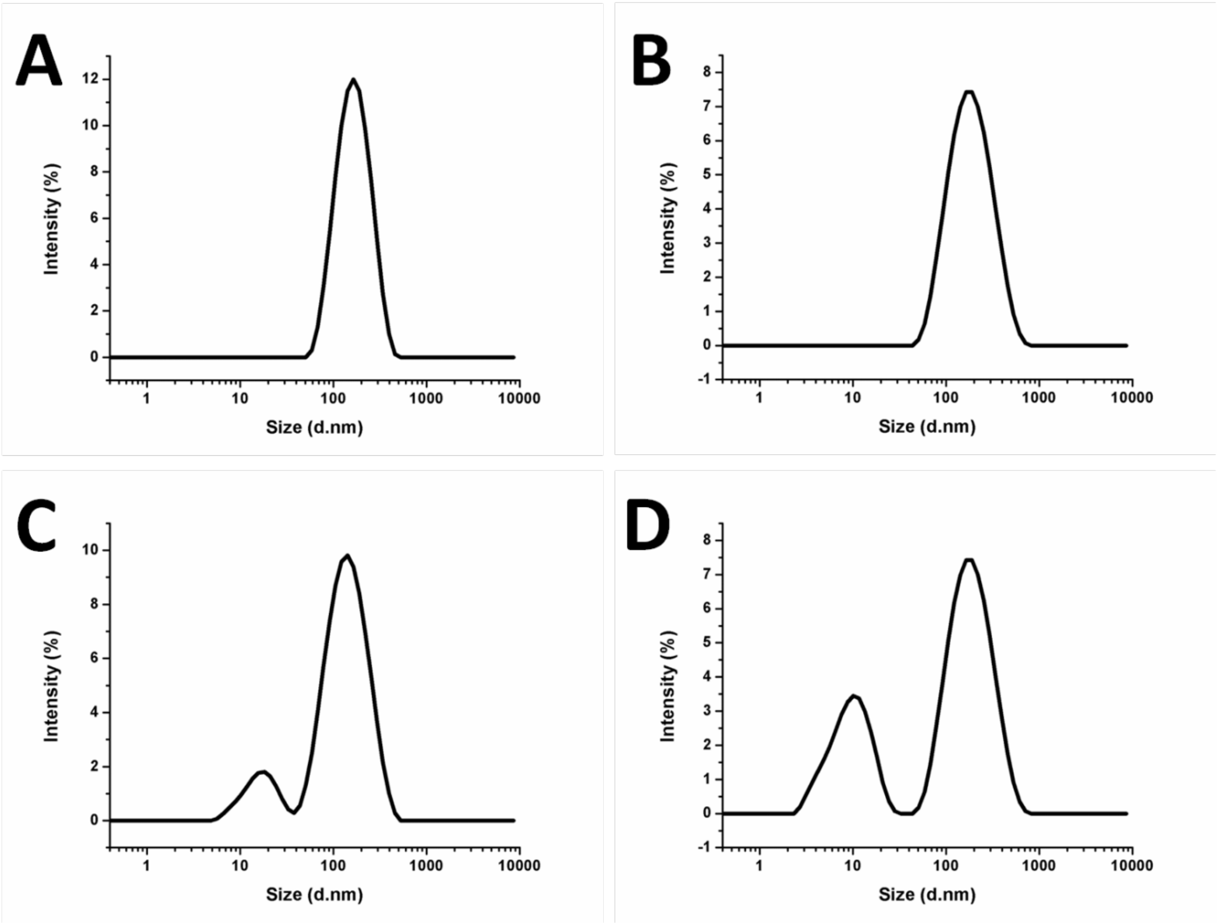

Figure S7. DLS size distribution of oxNG2-DFO incubated in ddH_2O after 24 h (**A**) and 240 h (**B**) reveals stable-sized nanoparticles; oxNG2-DFO incubated in 1% H_2O_2 (**C**) and 5% H_2O_2 (**D**) after 24 h reveal increasingly more degradation products in the presence of the oxidizer due to the oxidation-sensitive crosslinker. Comparatively, nanogels degrade faster when incubated at the higher concentration of H_2O_2.

References

[1] C. Feng, G. L. Lu, Y. J. Li, X. Y. Huang, *Langmuir* **2013**, *29*, 10922-10931.
[2] A. C. Feng, Q. Yan, H. J. Zhang, L. Peng, J. Y. Yuan, *Chem Commun* **2014**, *50*, 4740-4742.
[3] L. P. D. Ratcliffe, A. J. Ryan, S. P. Armes, *Macromolecules* **2013**, *46*, 769-777.
[4] M. Nakahata, Y. Takashima, A. Hashidzume, A. Harada, *Angew Chem Int Edit* **2013**, *52*, 5731-5735.
